# Foxo3a-mediated overexpression of microRNA-622 suppresses tumor metastasis by repressing hypoxia-inducible factor-1α in erk-responsive lung cancer

**DOI:** 10.18632/oncotarget.5826

**Published:** 2015-10-23

**Authors:** Chun-Wen Cheng, Po-Ming Chen, Yi-Hsien Hsieh, Chung-Chih Weng, Chia-Wei Chang, Chung-Chin Yao, Ling-Yueh Hu, Pei-Ei Wu, Chen-Yang Shen

**Affiliations:** ^1^ Institute of Biochemistry, Microbiology and Immunology, Chung Shan Medical University, Taichung, Taiwan; ^2^ Clinical Laboratory, Chung Shan Medical University Hospital, Taichung, Taiwan; ^3^ Department of Surgery, Chung Shan Medical University Hospital, Taichung, Taiwan; ^4^ Institute of Biomedical Sciences, Academia Sinica, Taipei, Taiwan; ^5^ College of Public Health, China Medical University, Taichung, Taiwan

**Keywords:** lung cancer, HIF-1α, EMT, miR-622, FOXO3a

## Abstract

Metastatic spread of cancer cells portends a poor prognosis and mortality for lung cancer patients. Hypoxia-inducible factor-1α (HIF-1α) enhances tumor cell motility by activating the epithelial-to-mesenchymal transition (EMT), which is considered a prerequisite for metastasis. Recent studies of microRNA involvement in cancer invasion and metastasis have highlighted the role of such RNAs in tumor development. However, little work has been done to identify tumor suppressor microRNAs that target HIF-1α to down-modulate the EMT and thereby counteract the aggressiveness and metastasis of lung cancer cells. Here, we identified the 3′-untranslated region of HIF-1α mRNA as a target of miR-622 and established that miR-622-mediated down-modulation of HIF-1α correlates with decreased levels of mesenchymal proteins, including Snail, β-catenin, and vimentin. Functional analyses revealed that increased miR-622 expression inhibited lung cancer cell migration and invasion *in vitro*. miR-622 also inhibited the genesis of metastatic lung nodules as demonstrated in a lung cancer xenograft model in which nude mice were transplanted with A549 cells expressing miR-622. Mechanistic analyses showed that overexpression of EGF decreased the miR-622 level in A549 cells, and this reduction could be rescued by administrating U0126, an inhibitor of ERK. Moreover, miR-622 overexpression mediated by the transcription factor FOXO3a decreased the invasiveness of lung tumor cells by inhibiting HIF-1α via inactivation of ERK signaling in U0126-treated A549 cells. These findings highlight the pivotal role of the FOXO3a/miR-622 axis in inhibiting HIF-1α to interfere with tumor metastasis, and this information may contribute to development of novel therapeutic strategies for treating aggressive lung cancer.

## INTRODUCTION

The initial malignant transformation of cells and subsequent metastasis are key steps in cancer progression. Developing new strategies that decrease the malignancy of cancer cells is therefore considered the most challenging issue facing therapeutic interventions to counter cancer mortality. Factors associated with increased tumor-specific angiogenesis, which is required to resolve hypoxia in tumor microenvironments, promote tumor expansion [1]. Studies have clearly demonstrated that hypoxia itself enhances pro-survival mechanisms underlying tumor outgrowth orchestrated by the activation of hypoxia-inducible factor-1 alpha (HIF-1α) encoded by *HIF-1* [2]. HIF-1α is essential for enabling angiogenesis and metastasis in a variety of solid cancers including lung cancer [3, 4]. The epithelial-to-mesenchymal transition (EMT), which can be induced by hypoxia [5], is considered to be a prerequisite for the typical tumor phenotypes of upregulated angiogenesis, enhanced cell motility, and extracellular matrix invasion. Regulation of the mesenchyme-specific transcription factor gene *Snail* (*SNAI1*), which is activated via the HIF-1α signaling cascade, enhances expression of the mesenchymal markers β-catenin and vimentin in hepatocellular carcinoma in response to hypoxia [6–8]. Importantly, HIF-1α is a key factor responsible for the transcriptional regulation of genes that facilitate the stemness properties of cancer cells and enhance their metastatic potential in leukemia and in prostate and breast carcinomas [9]. Accumulating lines of *in vitro* evidence indicate that HIF-1α is overexpressed in tumors to induce VEGF expression via activation of a signaling pathway downstream of the mitogen-activated protein kinase/extracellular signal–regulated kinase (MAPK/ERK) pathway [10, 11]. Thus, HIF-1α is an established target for the development of cancer therapeutics.

MicroRNAs (miRNAs) are small noncoding regulatory RNAs averaging 22 nucleotides in length that principally recognize target sequences of cognate mRNAs via less-than-perfect complementarity with the 3′-untranslated region (3′-UTR) of the mRNA, leading to cleavage of the target mRNA or repression of its translation [12, 13]. More than 30% of protein-coding genes are predicted to be regulated by miRNAs based on bioinformatic algorithms [14]. Intensive studies of lung cancer using gene expression profiling to investigate tumorigenesis and tumor progression have revealed that miRNAs function as tumor suppressors by negatively regulating oncogenes [15]. However, there has been scant identification of potent tumor suppressor miRNAs that target HIF-1α to down-modulate EMT and thereby counteract the aggressiveness and metastasis of lung cancer cells. Moreover, there have been even fewer attempts to retrieve critical molecular information regarding metastatic lung tumor cell–specific miRNA expression that may impact tumor progression. To address this deficiency, we predicted that the 3′-UTR of *HIF-1α* mRNA contains a sequence that directs miR-622-mediated translational repression, and indeed we validated *HIF-1α* mRNA as a target of miR-622. We thus used lentivirus-mediated transduction to establish two stable clones of the human lung cancer cell lines A549 and H1299 that express miR-622 to validate the ability of this miRNA to suppress cancer cell motility both *in vitro* and *in vivo*. We discovered a novel mechanism underlying miR-622-mediated regulation of *HIF-1α*, which is activated by the transcription factor FOXO3a. Our findings provide invaluable clues on how FOXO3a-mediated miR-622 overexpression regulates lung cancer progression, and this information may drive the optimization of the design and evaluation of potential miRNA-based therapeutics for metastatic lung cancer.

## RESULTS

### miR-622 represses HIF-1α expression by directly targeting the 3′-UTR of HIF-1α mRNA

**Figure 1:**
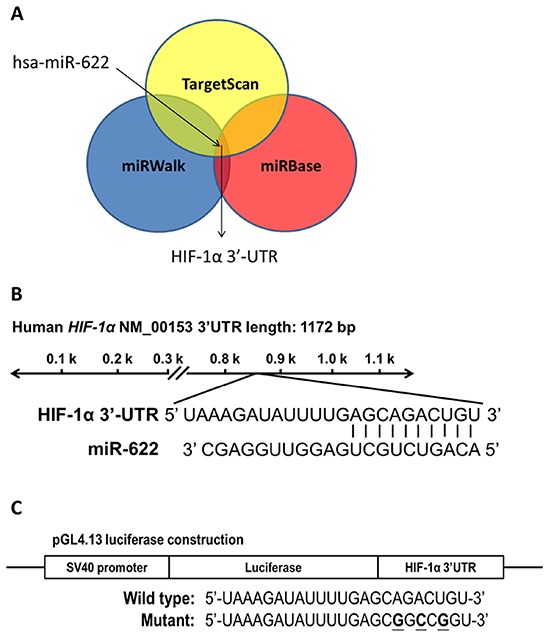

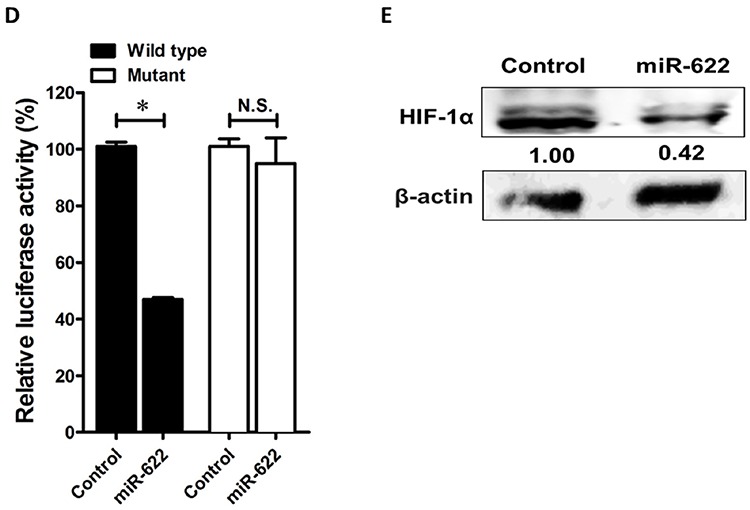
HIF-1α is a direct target of miR-622 **A.** Use of three algorithms demonstrates that miR-622 contains a sequence complementary to the 3′-UTR of HIF-1α. **B.** The predicted miR-622-target sequence is located at position 869–875 of the *HIF-1α* 3′-UTR in human chromosome 14q23.2. **C.** Schematic representation of the luciferase reporter constructs. The three bolded nucleotides represent mutant sites in the *HIF-1α* mRNA created by site-directed mutagenesis to yield mismatches with the complementary sequence in miR-622. **D.** A549-pLKO control cells (the negative control) and A549-pLKO/miR-622 cells were cultured in 24-well plates and transfected with 100 ng of wild-type or mutated HIF-1α 3′-UTR construct. The firefly luciferase/Renilla luciferase activity ratio of each sample was measured in a dual-luciferase reporter assay system. Each bar represents the mean ± S.D. of three independent experiments; *P < 0.05. **E.** HIF-1α level in A549-pLKO control cells and its reduced level in A549-pLKO/miR-622 cells overexpressing miR-622, as revealed by western blotting.

We used a combined computational prediction algorithm approach, including use of the programs miRBase (http://www.mirbase.org/), miRWalk (http://www.umm.uni-heidelberg.de/apps/zmf/mirwalk/), and TargetScan (http://www.targetscan.org/), to determine whether miR-622 contains a sequence complementary to the 3′-UTR sequence of *HIF-1α* (Figure 1A) on human chromosome 14q23.2 (Figure 1B). Toward this end, we used the pGL4.13-luciferase reporter to generate a construct encoding the full-length 3′-UTR of *HIF-1α* (wild-type *HIF-1α* 3′-UTR-luc) as well as a 3′-UTR/Mutant-luc with a mismatched version of the miR-622 complementary sequence (Figure 1C). We found that miR-622 significantly reduced the luciferase activity of the *HIF-1α* 3′-UTR-luc product by > 50%. Moreover, this reduction in activity was restored in the presence of the pGL4.13 reporter construct containing a mutation in the 3′-UTR of *HIF-1α* (Figure 1D). Furthermore, HIF-1α protein repression was more prominent in miR-622-transfected A549 lung cancer cells compared with control (Figure 1E). These results clearly demonstrated that miR-622 decreases HIF-1α expression by directly binding the 3′-UTR of its mRNA.

### miR-622 represses HIF-1α to inhibit invasiveness of lung cancer cells

**Figure 2:**
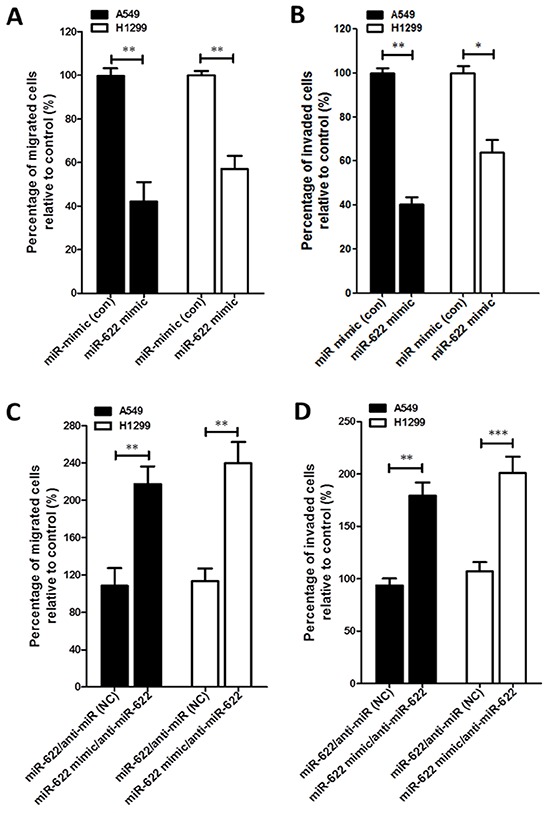

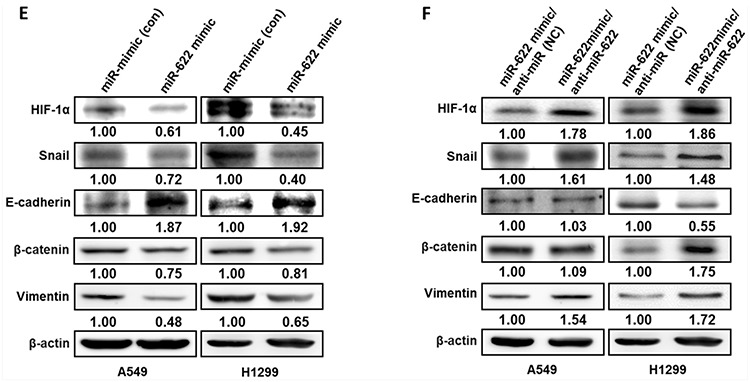
miR-622 inhibits the migration and invasion of lung cancer cells **A–D.** Boyden chamber assay for cells that were transiently transfected with a miR-622 mimic or mock transfected (miR-mimic, control). Cells were then plated on a porous membrane (8-μm diameter pore) coated without (A) or with (B) matrigel. Migration (C) and invasion (D) assays for miR-622-transfected lung cancer cells that had been treated with miR-622 inhibitor or negative control (anti-miR, NC). In panels A–D, cells in five random fields of view at 100× magnification were counted, and the results represent the mean ± S.D. of cells per field of view as measured in three independent experiments. *P < 0.05, **P < 0.01, ***P < 0.001. **E.** and **F.** Western blot for HIF-1α and EMT markers (Snail, E-cadherin, β-catenin, and vimentin) in A549 (left panel) and H1299 (right panel) cells transiently expressing miR-622 mimic or miRNA-mimic (control group, Con) in (E) and miR-622-transfected lung cancer cells treated with miR-622 inhibitor (anti-miR-622) or negative control (anti-miR, NC) in (F) β-actin was used as an internal control.

Tumor hypoxia induces EMT, which leads to invasion and metastasis by repressing the expression of the epithelial marker E-cadherin [16]. We therefore examined the suppressive function of miR-622 in lung cancer progression of two lung cancer cell lines *in vitro*. As expected, the migration and invasion abilities of miR-622-transfected A549 and H1299 lung cancer cells were diminished by >50% compared with mock-transfected controls (Figures 2A–2D and Supplementary Figure S1). In addition, miR-622 overexpression in these two cell lines reduced HIF-1α level, which resulted in decreased levels of Snail, β-catenin, and vimentin and an increased level of E-cadherin (Figures 2E and 2F).

**Figure 3:**
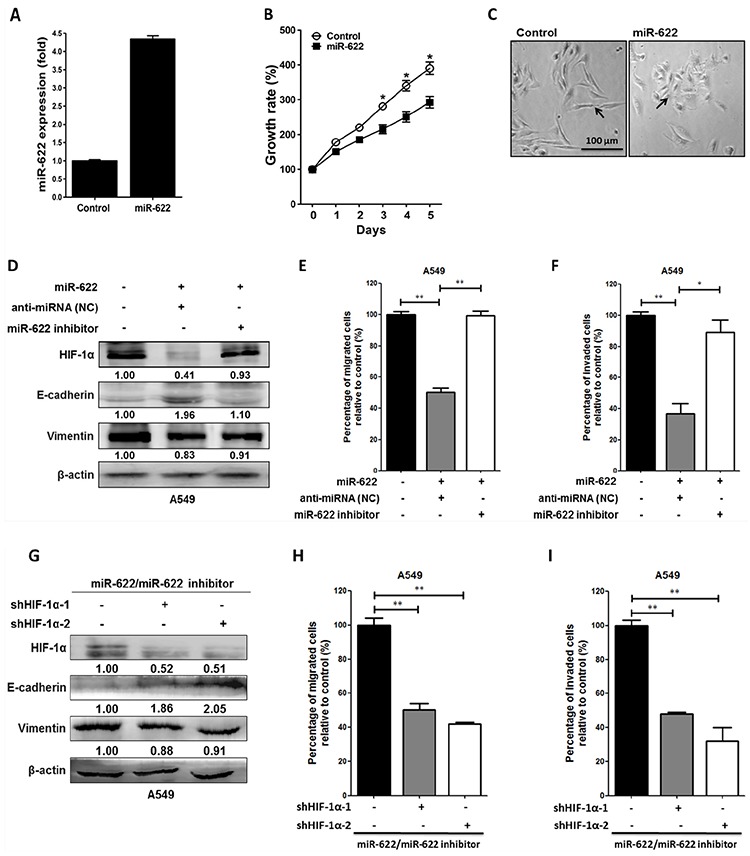
Overexpression of miR-622 inhibits the migration and invasion via repression of HIF-1α in lung cancer cells **A.** miR-622 mRNA expression level in A549 cells stably transfected with pLKO (Control) or A549-pLKO/miR-622 was quantified by reverse transcription-PCR (qRT-PCR). The small nuclear RNA *RNU6B* was used as an internal control. **B.** Proliferation of A549-pLKO (control) and A549-pLKO/miR-622 cells as assessed with the MTT assay. **C.** Representative images of the phenotypic change from a mesenchymal (A549/pLKO) to cobblestone-like epithelial state (arrow indicated) as seen in A549 cells overexpressing miR-622 (A549-pLKO/miR-622). **D.** Western blot for HIF-1α and epithelial (E-cadherin) and mesenchymal (vimentin) markers in A549 cells stably expressing pLKO/miR-622; the cells were treated with negative control anti-miRNA (negative control, NC) or miR-622 inhibitor (anti-miR-622). **E.** and **F.** Boyden chamber assay for detection of migration/invasion in A549 cells stably expressing miR-622; cells were treated with anti-miRNA (NC) or with miR-622 inhibitor RNA. **G, H, I.** Western blot (G) and Boyden chamber assay (H and I) for A549-pLKO/miR-622 cells that were transfected with shRNAs specific for HIF-1α (shHIF-1α-1 and shHIF-1α-2). Similarly, migration (H) and invasion (I) of cells were measured in five random fields of view at 100× magnification; cells were counted, and the results represent the mean ± S.D. of cells per field of view as measured in three independent experiments in panels (B), (E), (F), (H), and (I) *P < 0.05, **P < 0.01.

To underscore the contribution of miR-622 to the molecular mechanism that enables mesenchymal tumor cells to regain a cobblestone-like epithelial phenotype to inhibit cancer cell aggressiveness during the mesenchymal-to-epithelial transition of lung cancer cells, we used a lentivirus expression system and the Trans-Lentiviral™ Packaging System to establish a lentiviral vector pLKO stably expressing miR-622 transcripts. The A549-pLKO/miR-622-transfected cells had a ∼4.5-fold higher level of miR-622 compared with control (Figure 3A). After the third day of incubation, the proliferation rate of A549-miR-622 cells was significantly lower compared with control (Figure 3B). Interestingly, A549 cells with the increased miR-622 level underwent a morphological change from elongated and spindle-like fibroblasts to a cobblestone-like epithelial phenotype (Figure 3C). These data indicated that miR-622 overexpression in A549 cells altered cell morphology and diminished tumor cell proliferation as a consequence of the repression of HIF 1α expression. On the other hand, the decreased cancer cell motility upon miR-622 overexpression was restored upon transfection of A549 cells with an inhibitor against miR-622, and vimentin level was restored to that of the control (Figures 3D–3F). In parallel, results from HIF-1α silencing experiments with two short hairpin RNAs (shRNAs) targeting *HIF-1α* mRNA (shHIF-1α) revealed dramatic decreases in the migration and invasion of lung cancer cells (Figures 3G–3I), lending support to our theory that miR-622 inhibits tumor motility via repression of HIF-1α to down-modulate the EMT axis.

### miR-622 suppresses metastasis in a xenograft-transplantation model of lung cancer

**Figure 4:**
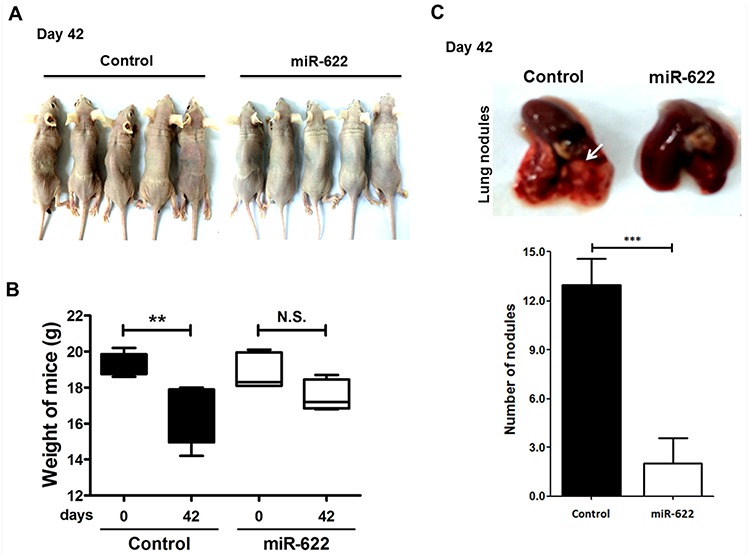

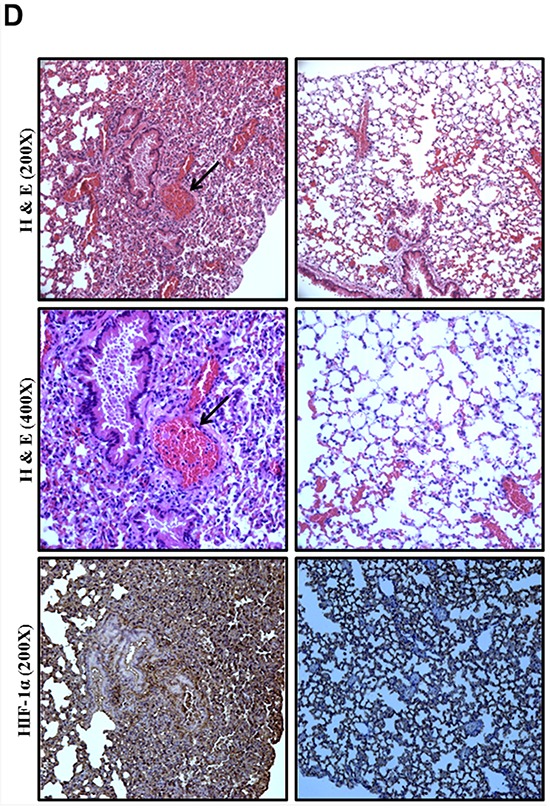
miR-622 inhibits the growth of xenografted lung cancer tumors **A.** Xenotransplantation studies were carried out in BALB/c null mice (*n* = 5 per group). **B.** Loss of body weight as measured on day 42 following tail-vein injection with 5 × 10^5^ A549-pLKO/miR-622 cells or A549-pLKO cells (control; *P* = 0.008). **C.** Number of metastatic tumor nodules in nude mouse xenografts at 42 days after injection with A549 cells stably overexpressing miR-622 (A549-pLKO/miR-622) or control cells with stably integrated empty vector (A549-pLKO) (upper panel). Data represent the mean ± S.D. for each group (lower panel). ***P < 0.001. **D.** Hematoxylin and eosin staining and immunofluorescence microscopy to detect HIF-1α in lung tissue sections bearing metastatic xenograft tumors with neovascularization (magnification with 200× and 400×) are shown. Arrows indicate vascularization of the lung tumor nodule.

Because we found that miR-622 plays a critical upstream mediator role in regulating lung cancer invasion and migration and repressing HIF-1α expression *in vitro* (Figure 2), we explored whether miR-622-associated metastatic suppression occurs *in vivo*. To examine whether miR-622 downregulates the HIF-1α axis with consequent effects on tumor metastasis, we established a xenograft model of human lung cancer cells in nude mice. In this model, A549 cells containing pLKO (control) or pLKO/miR-622 were injected into the tail vein (5 × 10^6^ cells/mouse). After 6 weeks, the average body weight in the control group was significantly reduced compared with the miR-622 overexpression group (Figures 4A and 4B). Mice were sacrificed and their lungs dissected to evaluate tissue morphology by hematoxylin and eosin staining. We found that mice injected with A549-pLKO/miR-622 cells had only a few pulmonary metastatic nodules on average and significantly fewer than the number of nodules formed in the control group (*P* < 0.001, Figure 4C). The control mice had significantly larger tumors and more extensive neovascularization, and the metastatic lung cancer tissues that lacked miR-622 overexpression had intensive staining for HIF-1α as assessed with immunohistochemistry (Figure 4D). This supported our hypothesis of a suppressive function for miR-622 in lung cancer metastasis *in vivo*.

### miR-622 is downregulated by ERK in lung cancer

**Figure 5:**
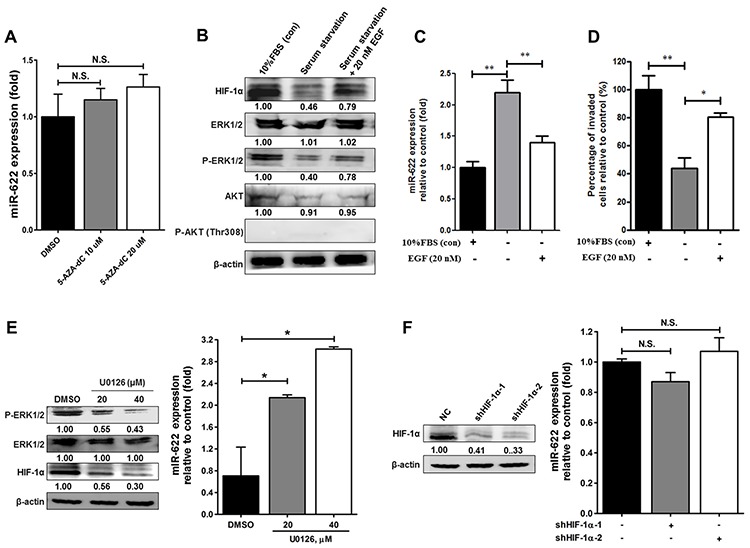
Effects of the EGF-ERK signaling pathway on the regulation of miR-622 expression in relation to invasiveness of lung cancer **A.** A549-pLKO cells were treated with 5-aza-2′-deoxycytidine (5-AZA-dC; 10 or 20 μM) for 72 h. qRT-PCR was used to determine miR-622 level normalized to untreated cells. N.S., not significant. **B.** Western blotting for HIF-1α, ERK, phosphorylated ERK1/2 (p-ERK1/2), AKT, and phosphorylated AKT at T308 [p-AKT (Thr308)] in A549-pLKO/miR-622 stably expressing cells under serum starvation or restored by treatment with either 10% FBS or EGF (20 nM) for 6 h in a hypoxic state as described in Materials and methods. **C.** Quantitative results for the miR-622 level in A549-pLKO/miR-622 cells treated with EGF (20 nM) or 10% FBS was analyzed by qRT-PCR. *RNU6B* was used as an internal control. **D.** Boyden chamber assay for the assessment of invasion capacity of A549-pLKO/miR-622 cells treated with EGF (20 nM) or 10% FBS for 12 h. The results presented in panels (C) and (D) represent the mean ± S.D. of three independent experiments. *P < 0.05, **P < 0.01. **E.** Western blot for HIF-1α, ERK, and phosphorylated ERK1/2 (p-ERK1/2), (left panel), and qRT-PCR analysis of miR-622 level (right panel) in A549 cells stably expressing miR-622; cells were treated with the ERK kinase inhibitor, U0126, for 24 h. **F.** Western blot and qRT-PCR detection of HIF-1α expression in A549-pLKO/miR-622 cells that had been transfected with HIF-1α shRNAs (shHIF-1α-1 and shHIF-1α-2). N.S., non-significant.

miRNA genes are frequently downregulated via promoter hypermethylation in different types of solid cancers [17–20]. We therefore examined whether the observed reduction in miR-622 level in lung cancer cells was ascribable to DNA hypermethylation. However, we found no significant change in miR-622 level in A549 cells treated with 5-aza-2′-deoxycytidine (Figure 5A). Alternatively, evidence has shown that induction of angiogenesis by HIF-1α in cancer cells is activated by epidermal growth factor (EGF)/AKT/ERK signaling during hypoxia [21], and our analysis of miR-622-overexpressing cells revealed decreased HIF-1α-dependent cell invasion. We therefore hypothesized that lung cancer cell aggressiveness could be rescued by upregulating EGF/AKT/ERK signaling to compensate for the miR-622-mediated downregulation of HIF-1α. Support for our hypothesis came from our observation that A549 cells treated with 10% serum or EGF (20 nM) had a reduced level of miR-622 (Figure 5C); this lower miR-622 level led to invasiveness (Figure 5D) via increased HIF-1α expression as a consequence of ERK phosphorylation in the EGF-treated A549 cells (Figure 5B). This inhibitory effect on miR-622 level was comparable to that of the ERK inhibitor, U0126, and inactivation of phosphorylated ERK in the lung cancer cells resulted in a higher miR-622 level in a dose-dependent manner (Figure 5E). Notably, monitoring phosphorylation of Akt at T308 has been shown to improve the assessment of Akt activation and revealed that Akt activation is a poor prognostic factor for lung cancer [22], we thus examined the activation of AKT in association with miR-622 expression level during hypoxia; however, T308 of AKT kinase was not phosphorylated in response to EGF treatment in A549 cells (Figure 5B). In addition, although phosphorylation of ERK reduced the miR-622 level, miR-622 level was not affected by *HIF-1α* knockdown in A549 cells (Figure 5F). We concluded that a block of EGF/ERK signaling may inhibit lung cancer metastasis via miR-622-mediated repression of the HIF-1α axis, which, as a consequence, precludes feedback inhibition of miR-622 level by HIF-1α.

### FOXO3a upregulates miR-622 level

**Figure 6:**
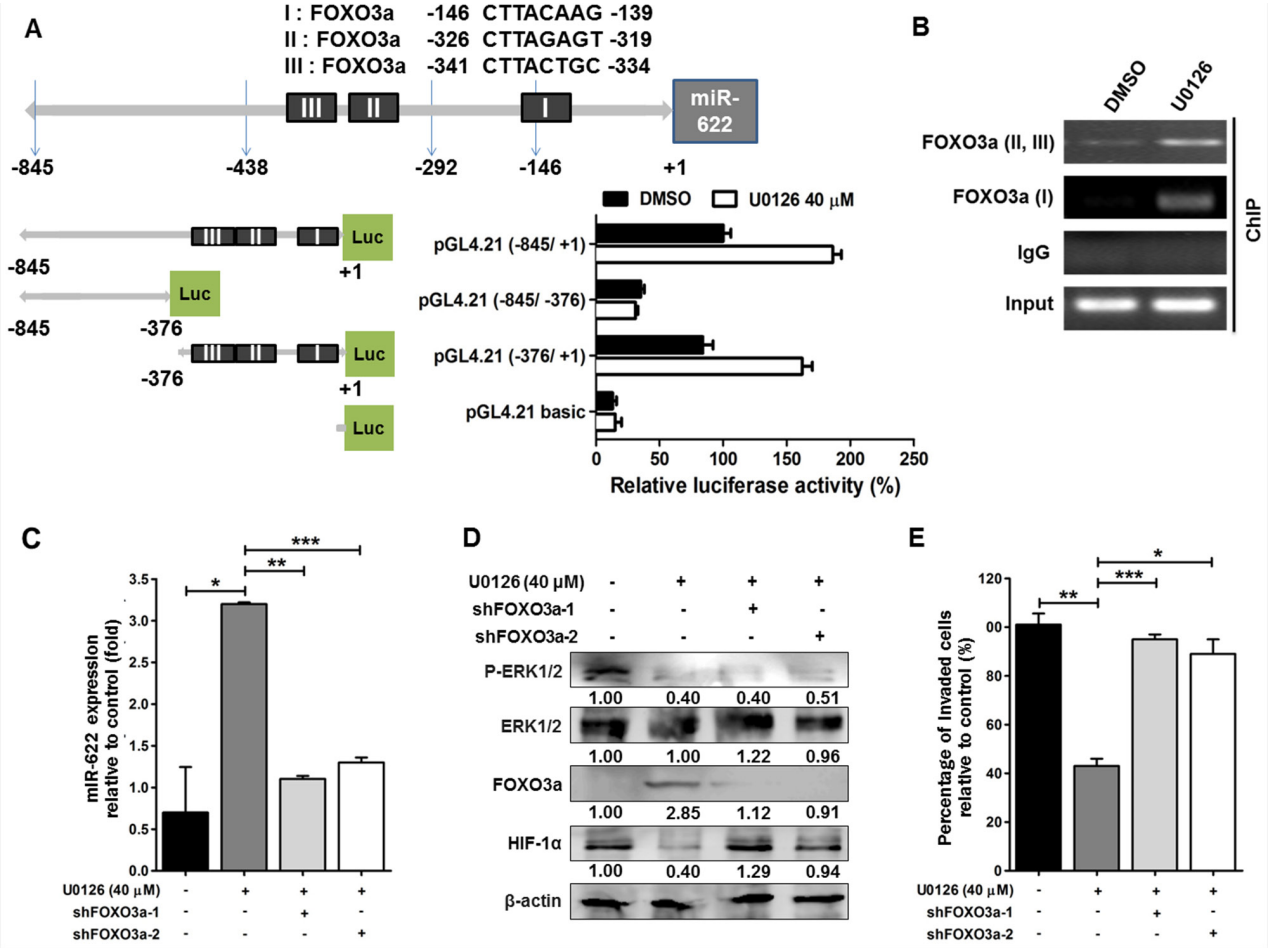
Expression of the miR-622 gene is activated by FOXO3a **A.** Analysis of the miR-622 promoter. Schematic representation of the different putative miR-622 promoter regions were inserted upstream of the pGL4.21-basic luciferase gene (upper panel). A549 cells were transfected with the various vectors, and the luciferase activity was measured (lower panel). **B.** The interaction between FOXO3a and the miR-622 promoter region I (nt −845 ∼ +1) or II (nt −376 ∼ +1) in A549 cells was detected by chromatin immunoprecipitation (ChIP) with a FOXO3a-specific antibody or IgG (control). DMSO, dimethylsulfoxide. **C.** Western blotting for FOXO3a, HIF-1α, ERK1/2, and p-ERK1/2 in A549 cells treated with shFOXO3a-1 or shFOXO3a-2 in the presence of ERK inhibitor U0126. **D.** qRT-PCR was used to measure miR-622 level in A549 cells upon FOXO3a knockdown using shFOXO3a-1 or shFOXO3a-2; cells were treated with U0126. **E.** Boyden chamber assay for assessment of the invasiveness of shFOXO3a-A549 cells after treatment with U0126. Data represent the mean ± S.D. of three independent experiments. *P < 0.05, **P < 0.01, ***P < 0.001.

Inhibition of FOXO proteins by ERK depends on ERK phosphorylation that leads to ubiquitination of FOXOs and their subsequent degradation in proteasomes [23]. Based on bioinformatics, we found that the primary miRNA transcript of miR-622 (pri-miR-622) is located at chromosome 13q31.3, and an *in silico* analysis with ALLGGEN_PROMO predicted three putative FOXO3a-binding sites in the pri-miR-622 promoter region; thus, we speculated that FOXO3a might regulate miR-622 expression. To test this possibility, we designed a FOXO3a knockdown experiment in combination with altered pri-miR-622 promoter constructs. In the gene encoding miR-622, three fragments upstream of the 5′-UTR of miR-622 gene were amplified and cloned into the vector pGL4.21-Basic-Luc; these were named (−845/+1)-Luc, (−845/–376)-Luc, and (−376/+1)-Luc (Figure 6A, upper panel). In the presence of U0126 (40 μM), A549 cells having either promoter construct (−845/+1)-Luc or miR-622 (−376/+1)-Luc showed an approximate 2-fold increase in luciferase activity compared with the negative control (Figure 6A, lower panel). Additionally, chromatin immunoprecipitation assays performed with an antibody against FOXO3a showed that FOXO3a binds the upstream promoter region of the miR-622 gene in response to ERK (Figure 6B). Further, because U0126-mediated ERK inactivation in A549 cells stabilized endogenous FOXO3a mRNA (Supplementary Figure S2A) and protein (Supplementary Figure S2B), we designed a FOXO3a knockdown experiment in U0126-treated A549 cells and found that the effect of FOXO3a on miR-622 elevation was abrogated by shFOXO3a (a FOXO3a-specific shRNA; Figure 6C). Concomitantly, upon ERK inactivation, the decreased level of HIF-1α in the miR-622-overexpressing A549 cells was restored upon transfection with either of two shFOXO3a constructs (Figure 6D), which also resulted in enhanced tumor cell invasiveness compared with control oligonucleotide-transfected cells (Figure 6E). Taken together, our data suggested that the inhibitory effect of FOXO3a-mediated miR-622 expression on the decrease in invasion capacity of cancer cells is controlled by suppressing ERK-HIF-1α signaling.

### ERK activation downregulates the FOXO3a-miR-622 axis to increase HIF-1α expression in lung cancer cell invasion

**Figure 7:**
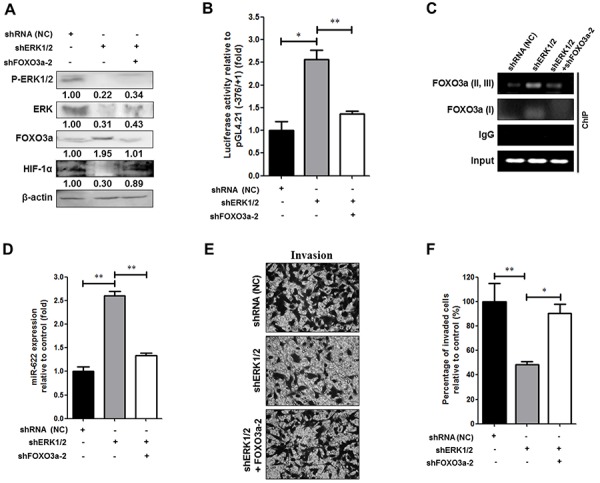
Phosphorylated ERK downregulates FOXO3a-miR-622 to increase HIF-1α expression and promote the invasiveness of human lung cancer cells **A.** Western blotting to detect ERK, p-ERK1/2, FOXO3a, and HIF-1α in A549-pLKO/miR-622 cells that were transfected with the following shRNAs: negative control shRNA (NC), shERK1/2, or shFOXO3a-2, as indicated. **B.** A549-pLKO/miR-622 cells transfected with pGL4.21 containing the −376/+1-Luc reporter construct along with shREK1/2, shFOXO-3a, or shRNA (negative control, NC), as indicated, were incubated for 24 h and then harvested to assess relative luciferase activity (mean ± S.D.) **C.** An interaction between FOXO3a and the two miR-622 promoter regions I (nt −845 ∼ +1) and II (nt −376 ∼ +1) in A549 cells was detected by ChIP with a FOXO3a-specific antibody or IgG (control) as detected by transfection of A549 cells with shERK1/2, shFOXO3a, or NC shRNA. **D.** qRT-PCR analyses of miR-622 level in A549 cells transfected with different shRNAs as indicated in (A). **E.** Representative photos of Boyden chamber assay, and **F.** invasion capacity was assessed in A549 cells transfected with different shRNAs as indicated in (A) Results represent the mean ± S.D. of three independent experiments in panels (B), (D), and (E) *P < 0.05, **P < 0.01.

As shown in Figure 6, FOXO3a upregulates miR-622 to repress HIF-1α expression via inactivation of ERK phosphorylation, resulting in the inhibition of the invasiveness of A549 cancer cells. Further, we utilized a luciferase gene construct encoding the promoter region of HIF-1α (pGL4.21 (−376/+1), shown in Figure 6A); the resultant promoter activity values showed that the normalized luciferase activity increased in shERK1/2-treated cells compared with control shRNA cells. By contrast, this increase in luciferase activity was reduced by > 60% in shERK1/2-cells treated with shFOXO3a (Figures 7A and 7B). In addition, we used chromatin immunoprecipitation with an antibody against FOXO3a; the DNA fragment was captured in A549 cells transfected with shERK1/2, whereas this was not the case for cells treated with shFOXO3a or shRNA negative control (Figure 7C). We also found that the miR-622 level was significantly higher in A549 cells treated with shERK1/2 (Figure 7D) compared with cells treated with the negative-control shRNA, and the miR-622 level in shFOXO3-treated cells was much lower than that in shERK1/2-treated cells, indicating that miR-622 downregulation correlates with the ERK-FOXO3a axis. Likewise, we confirmed that the number of invading cells was significantly reduced upon shERK1/2 treatment (Figure 7E). Moreover, this decrease in invasiveness of A549 cells transfected with shERK1/2 was restored upon transfection with shFOXO3a, again confirming the role of the FOXO3a-miR-622 axis in repressing HIF-1α expression to account for ERK activation and its consequent upregulation of the invasiveness of human lung cancer cells (Figure 7E and 7F).

## DISCUSSION

Downregulation of miR-622 promotes cellular invasion and metastasis in different types of cancers, including brain, esophageal, and gastric cancer [24–26], but the molecular mechanism involved in lung cancer metastasis and the target gene modulated by miR-622 remain unknown. In the hypoxic microenvironment of solid tumors, HIF-1α expression in certain clones affords a selective advantage through multiple mechanisms that rely on angiogenesis and EMT, which ultimately increases tumor aggressiveness. HIF-1α overexpression in lung tumor cells is associated with increased invasion capacity and metastasis, and HIF-1α serves as a biomarker of poor prognosis in human lung cancer [27]. The most prescient findings of the present study are that miR-622 directly targets HIF-1α as assessed with three algorithms and that the resultant repression of HIF-1α inhibits cancer cell migration and invasion as assessed *in vitro* in two lung tumor cell lines under hypoxia. Our xenotransplantation study of the tumorigenicity of human lung cancer cells in mice confirmed that the number of lung nodules was significantly decreased in animals that received cells overexpressing miR-622, which facilitated downregulation of HIF-1α level. These findings not only describe a relationship between the miR-622-mediated suppression of lung cancer metastasis (based on targeting HIF-1α) but also delineate the mechanism underlying the inhibitory effect of miR-622 on lung cancer progression via repression of the transcription factor Snail, which is responsible for the mesenchymal phenotype observed during EMT.

**Figure 8:**
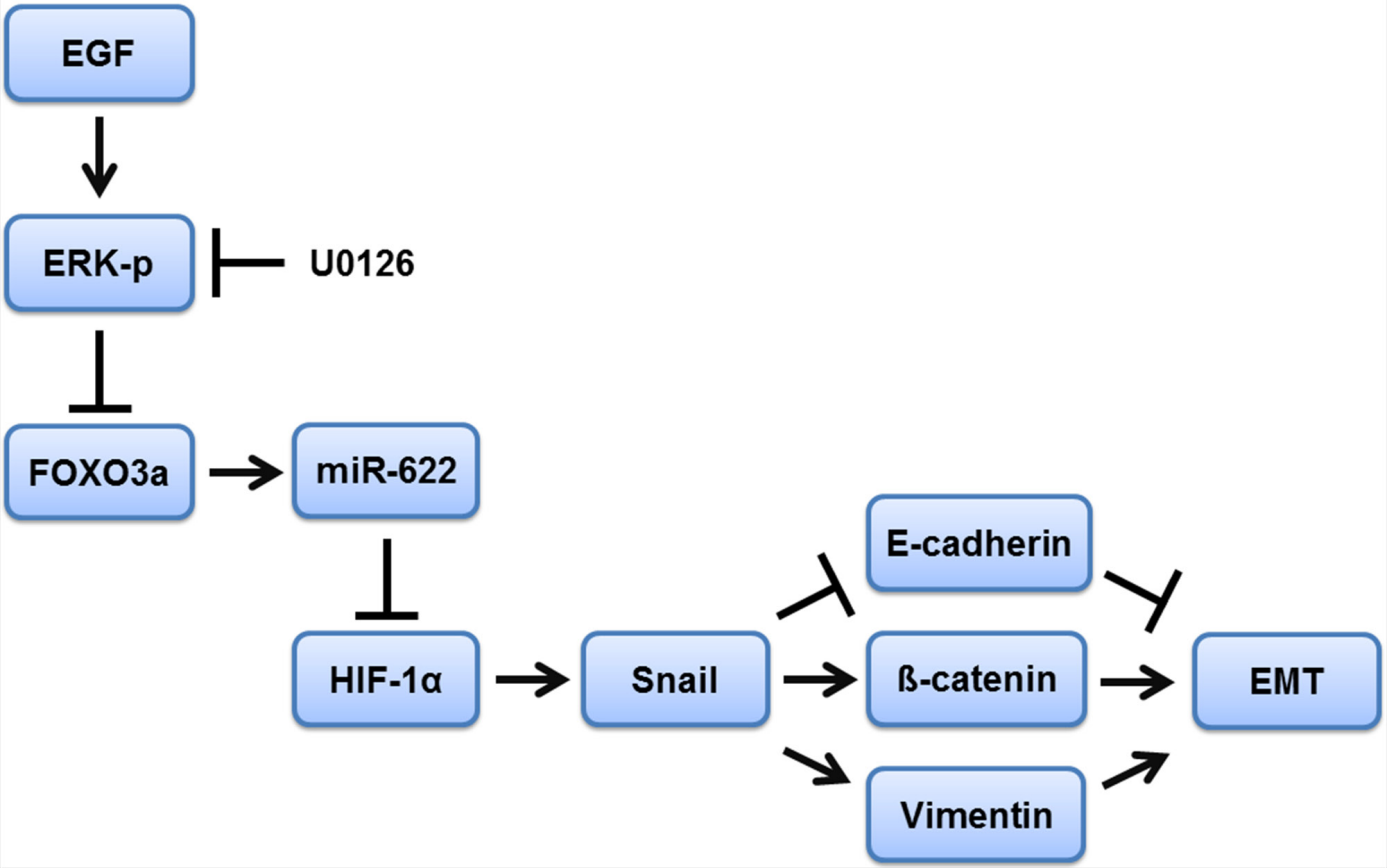
Schematic representation of FOXO3a-mediated overexpression of microRNA-622 suppresses tumor metastasis by repressing HIF-1α in ERK-responsiveness of lung cancer A blockade of EGF/ERK signaling enhances FOXO3a-induced miR-622 transcription, which inhibits the HIF-1α-EMT axis and leads to diminished tumor invasion and metastasis in lung cancer.

Recent studies have shown that miRNA expression in tissues is regulated by the methylation of DNA sequences upstream of the corresponding miRNA gene and that cancer cell DNA is frequently hypermethylated compared with normal cells [28–30]. We conjectured that this modification could be involved in miR-622-mediated silencing in lung cancer, and thus we investigated global DNA methylation patterns of the miR-622 promoter in A549 cells. Our results, however, showed that the reduced level of miR-622 in lung cancer did not correlate with DNA hypermethylation in the miR-622 promoter sequence. In contrast, expression levels of certain miRNAs are tightly regulated during the cellular response to hypoxia in poorly differentiated solid tumors, and indeed EGF acts as an inhibitor to decrease the levels of tumor-suppressor miRNAs [31–33]. Intriguingly, we found a correlation between EGF treatment and miR-622 reduction under hypoxia, which led to an elevated HIF-1α level and promoted tumor cell invasion (Figure 5). Hypoxia upregulates the EGF receptor and prolongs the activation of ERK and AKT signaling, which contributes to tumor-related angiogenesis and tumorigenesis [23, 34, 35]. Our present data show that, although treatment of A549 cells with EGF did not activate the kinase AKT, it enhanced the phosphorylation of ERK, which led to a decrease in FOXO3a level. Notably, blocking ERK activation with U0126 enhanced FOXO3a-mediated miR-622 transcription that resulted in inhibition of HIF-1α expression in cancer cells, leading to decreased tumor invasion and metastasis (Figure 8).

Going beyond the current study of the inhibitory effects of miR-622 on lung cancer progression owing to HIF-1α reduction, our results suggest a mechanism underlying down-modulation of HIF-1α by miR-622 that then inactivates EMT pathway genes, leading to reduced levels of Snail, β-catenin, and vimentin. In response to hypoxia, HIF-1α and a broad array of its downstream targets are synthesized *de novo* as a consequence of defects displayed by a variety of tumor suppressors in concert with organ-specific cancer cell invasion and migration. For example, deficiency of von Hippel-Lindau function counters the degradation of HIF-1α under normoxia [16, 36], and HIF-1α represses the transcription of the E-cadherin gene, contributing to EMT in von Hippel-Lindau-null renal cell carcinomas [16, 37]. In brain and breast cancers, HIF-1α overexpression increases angiogenesis by upregulating the levels of VEGF, interleukin-8, and basic fibroblast growth factor [38]. Recent association studies between miRNA markers and lung cancer development have demonstrated that the miRNAs miR-18, miR-199, and miR-519c can suppress HIF-1α expression for the purpose of assessing cancer prognosis [39–41]. Therefore, it would be informative to validate additional genetic factors, including miRNAs, that are involved in modulating HIF-1α level in collaboration with mesenchymal markers to potentially help predict risk of aggressive lung cancer.

MiRNAs post-transcriptionally regulate the expression of hundreds of tumor-suppressor genes that control a wide range of biological and physiological events, leading to inhibition of tumorigenesis and cancer progression as well as the promotion of tumor cell death in different cancer types [42, 43]. To our knowledge, the present study is the first to suggest the potential prognostic significance of FOXO3a-mediated miR-622 transcription that then downregulates HIF-1α and decreases tumor aggressiveness in response to EGF-activated ERK signaling in lung cancer. Our data demonstrate a role for miR-622 in repressing metastasis via inhibition of HIF-1α-related EMT signaling and thus suggest that miR-622 could be utilized as a promising target for the development of a lung cancer therapeutic.

## MATERIALS AND METHODS

### Cell lines, virus generation, and infection

The lung cancer cell lines A549 and H1299 were obtained from the American Type Culture Collection (Manassas, VA, USA) and cultured in Dulbecco's modified Eagle's medium (DMEM; Life Technologies) containing 0.1 mM sodium pyruvate, 10% FBS, 2 mM l-glutamine, 100 IU/mL penicillin, and 100 μg/mL streptomycin. For the incubation of cells under conditions that mimic hypoxia, cells were treated with deferoxamine mesylate (Sigma-Aldrich, St. Louis, MO, USA) or cultured at 37°C in a hypoxia chamber (1% O_2_, 5% CO_2_, 94% N_2_ atmosphere). Cells were transfected using Lipofectamine™ 2000 (Invitrogen, Carlsbad, CA, USA). A549 cells were maintained in DMEM with 10% FBS. Briefly, 2 × 10^6^ HEK293T (human embryonic kidney) cells were cotransfected with 10 μg of the lentiviral vector pLKO (control) or pLKO/miR-622, 9 μg of pCMVΔR8.91 (packaging plasmid), and 2.5 μg of pMDG (envelope plasmid), which were purchased from the National RNAi Core Facility at Academic Sinica, Taiwan. At 24 h post-transfection, virus-containing supernatants were collected. Lentiviral infection was performed by adding virus-containing supernatant to A549 cells (1 × 10^6^) at the desired multiplicity of infection in the presence of 8 ng/mL polybrene. After 48 h, stable transfectants were selected under 5 μg/mL puromycin. miR-622 expression was confirmed by real-time PCR.

### Reverse transcription and real-time PCR

Primers specific for miR-622 and random hexamers (supplied with the TaqMan miRNA assay) were obtained from Applied Biosystems. Real-time PCR was performed using an Applied Biosystems 7000 Fast Real-time PCR system with miR-622 primers and TaqMan Universal PCR Master Mix and AmpErase UNG (uracil-N-glycosylase; Applied Biosystems). Values represent the average of three independent experiments, normalized to the endogenous control gene *RNU-6B*.

### Cell proliferation assay

Lung cancer cells (2 × 10^3^ cells) were cultured in 96-well flat-bottomed microtiter plates supplemented with DMEM containing 10% heat-inactivated FBS, 100 U/mL penicillin, and 100 U/mL streptomycin in a humidified atmosphere of 95% air and 5% CO_2_ at 37°C. Cell viability was determined with the MTT (methyl thiazolyl tetrazolium) assay (absorbance read at 570 nm), and cell viability is expressed as a percentage of viability measured for the relevant control cells.

### Western blotting

The detailed procedure for western blotting has been described [44]. Western blotting used primary antibodies (diluted 1:1000) against the following proteins: AKT (sc-8312), phosphorylated AKT (sc-16646-R), ERK (sc-94), phosphorylated ERK (sc-7383), and vimentin (sc-6260); each antibody was purchased from Santa Cruz Biotechnology (Santa Cruz, CA, USA). Antibodies against HIF-1α (Cell Signaling) and E-cadherin (cat. 610182, BD Biosciences, Franklin Lakes, NJ) were also used. Proteins separated by SDS-PAGE were transferred to a Hybond-C Extra membrane (GE Healthcare, Little Chalfont, UK) that was then subjected to western blotting with an appropriate primary antibody. Anti-mouse or anti-rabbit IgG conjugated to horseradish peroxidase was used as the secondary antibody for detection using an ECL western blot detection system (Millipore, Bedford, MA, USA), and band intensities were quantified by densitometry (Digital Protein DNA Imagineware, Huntington Station, NY).

### Site-directed mutagenesis

Site-directed mutagenesis was performed to generate the mutant HIF-1α 3′-UTR sites of the luciferase construct using complementary oligos (Figure 1). Plasmids containing multiple point mutations within the sites were generated using the QuikChange site-directed mutagenesis system (Stratagene, Santa Clara, CA). Different concentrations of expression plasmids were transiently transfected into lung cancer cells (1 × 10^6^) using Transfast reagent (Thermo, Waltham, MA). After 48 h, the cells were harvested, and whole-cell extracts were assayed in subsequent experiments.

### Dual luciferase reporter assay

The 3′-UTR sequence of human *HIF-1α* was cloned into plasmid pGL4.13 (Promega Co., Madison, WI, USA) to produce the recombinant vector pGL4.13/*HIF-1α* 3′-UTR wt, which contains the firefly luciferase open reading frame under the control of the SV40 promoter. The mutated nucleotides are underlined, and the sequences of the mismatch primers used to generate the different *HIF-1α* 3′-UTR mutants are shown in Figure 1C. A549 cells were cultured in 24-well plates and co-transfected with 100 ng of *HIF-1α* wild-type or mutated 3′-UTR construct and pLKO (the negative control) or pLKO/miR-622. The firefly luciferase/Renilla luciferase activity ratio of each sample was measured in the dual-luciferase reporter assay system (Promega Co., Madison, WI, USA).

### Flow cytometry

Cells (5 × 10^5^) were seeded onto 6-cm dishes and cultured at 37°C for 24 h. The cell pellets were washed twice with PBS and fixed with 70% ethanol at 4°C for 30 min. After centrifugation at 200 × *g* for 5 min, the cell pellets were washed with PBS to remove any residual ethanol. Finally, the cells were resuspended in 1 mL of solution containing 0.5 mg/mL RNase A, 1% (w/v) Triton X-100, and 40 μg/mL propidium iodide and incubated at 37°C for 30 min. The cells were filtered through a 40-μm nylon mesh before flow cytometry analysis of cell-cycle distribution using a FACSCalibur flow cytometer (BD Biosciences).

### Migration/invasion assay

Cells were trypsinized and collected from culture dishes. Samples consisting of 5 × 10^4^ cells were seeded into 24-well modified Boyden chambers with polycarbonate membranes (8 μm pore size) to evaluate their migration (without Matrigel) and invasion (with Matrigel) capabilities for 12 h.

### Xenograft tumor formation

All mice were housed in the animal facility at the Chung Shan Medical University Experimental Animal Center, Taichung, Taiwan. Ethics approval was obtained for the use of animals, and all experiments were performed in accordance with the guidelines for animal care of the Institutional Animal Care and Use Committee of Chung Shan Medical University. Five-week-old female immunodeficient nude mice (BALB/c nu/nu) were injected with A549-pLKO or A549-pLKO/miR-622 cells via the tail vein (5 × 10^5^ cells in 0.1 mL of PBS). After 42 days, mice were sacrificed by CO_2_ asphyxiation. The number of metastatic lung tumors was confirmed with hematoxylin and eosin staining under a dissecting microscope.

### Statistical analysis

Statistical significance of the experimental data grouped by one variable was assessed by the unpaired two-tailed Student's *t*-test, one-way ANOVA, or Dunnett's test as appropriate. All statistical analyses were performed using SPSS version 17.0 (SPSS Inc., Chicago, IL, USA). A value of *p* < 0.05 was considered to indicate statistical significance.

## SUPPLEMENTARY FIGURES


