# Supplementary material for: Foxo3a-mediated overexpression of microRNA-622 suppresses tumor metastasis by repressing hypoxia-inducible factor-1α in erk-responsive lung cancer
Source: Oncotarget. 2015 Oct 23;6(42):44222–38. doi: 10.18632/oncotarget.5826 (PMC4792553; doi:10.18632/oncotarget.5826)
Supplement: Supplementary file 1 [file oncotarget-06-44222-s001.pdf]

SUPPLEMENTARY FIGURES

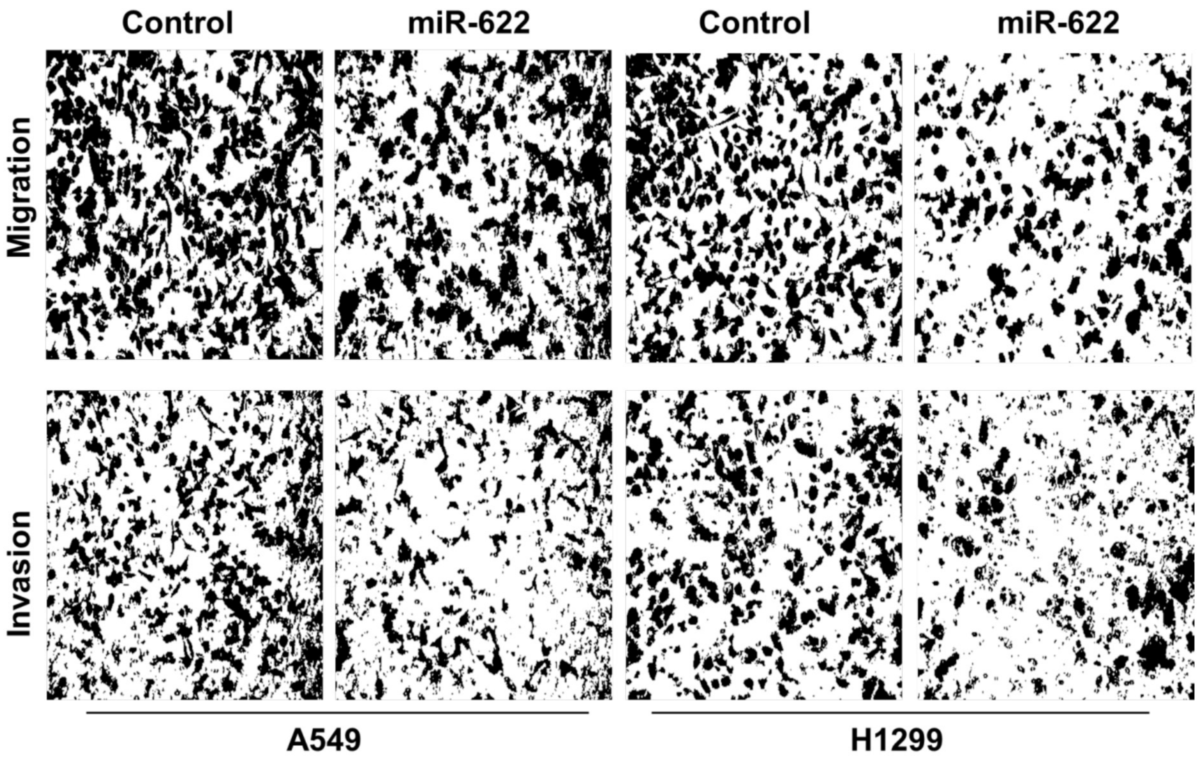

Supplementary Figure S1: Ectopic expression of miR-622 inhibits migration and invasion in lung cancer. Boyden chambers with polycarbonate membranes (8 μm pore size) were used to evaluate cell migration (without Matrigel) and invasion (with Matrigel). The migration and invasion capacities of A549 (left panel) and H1299 (right panel) lung cancer cells were diminished in cells transfected with miR-622 compared with mock-transfected control cells.

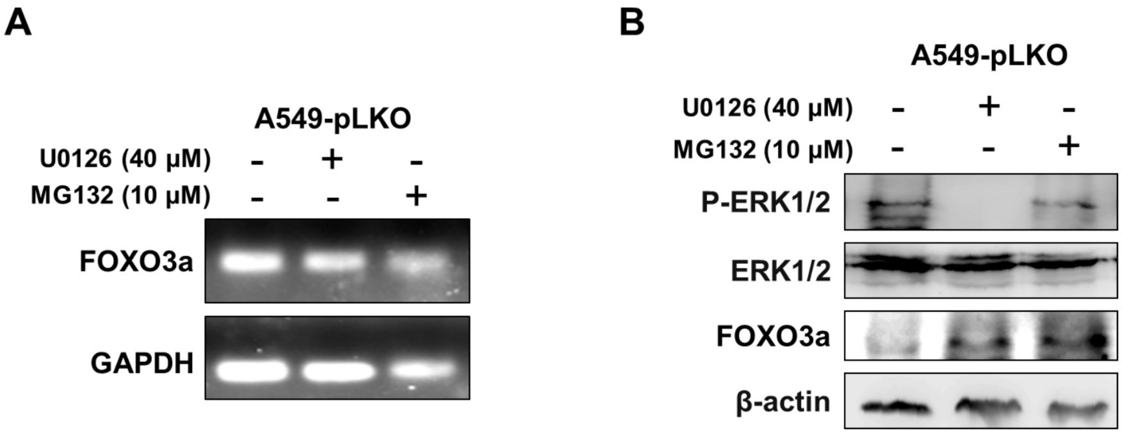

Supplementary Figure S2: FOXO3a expression is regulated by ERK. A. ERK inactivation mediated by U0126 in A549 cells stabilizes endogenous FOXO3a mRNA. GAPDH served as the reference. **B.** Phosphorylation of FOXO3a by ERK targets p-FOXO3a for proteasomal degradation. In contrast, FOXO3a was stabilized in A549 cells treated with the proteasome inhibitor MG132.
